# The Open Coal-Fire

**Published:** 1919-09-20

**Authors:** 


### HOSPITAL CONSTRUCTION: THE NEW ERA.

### XI. The Open Coal-fire.

If the Englishman's house is his castle, the open coal-fire is, by a slight straining of metaphor, the very banner upon his ramparts—the sign and emblem of his baronial domesticity. Possibly the Briton's home of the near future may, under the taint of State aid, lose something of its character as a mediæval fortress, and with that lessening of the majesty of home there may come also a lowering of the prestige of the liberal, the profligate, coal-fed hearth. Its death will come as a sad day for England. The survival of this cheerful extravagance has been a pleasant sign of England's illogical economy, and those who love the traditional features of our ancient civilisation will mourn with unfeigned grief the departure of this friendly centre of insular idolatry. So in our homes let us hope and pray for the continued long life of the British hearth.

But what of the coal-fire in the ward? Its advocates are many and staunch. Patients love it, nurses adore it, and doctors say that it produces a ventilation which no other system of heating can equal or rival.

Can these supporters of the fire of coals hold their ground. Let us consider the case. A good-sized ward fireplace, suitable for effecting the heating of the space occupied by ten patients, involves the initial expenditure, at present prices, of the following sums:—£5 for a grate, £10 for a chimney-piece, a hearth and tiles costing, let us say, £2, and brickwork to the average bulk of 65 cubic feet, which we must put at £9. Here to begin with is a total in initial expenditure of some £26. To this we must add the consideration that the chimney-breast occupies about 7½ square feet of floor-space and robs the air-space of the ward of about 90 cubic feet. This is not the whole of the indictment. These fireplaces must either be placed against one or more of the four walls of the ward, or they must come in the centre of the room. In the former case they either occupy the space which might be taken by a bed, or they rob us of a window. In the latter, two courses are possible: we either construct a flue above them, which means the existence of a column of brickwork that sadly hampers the inspection of the ward and is consequently a bar to easy supervision, or we contrive one of those quasi-miraculous arrangements by which the smoke, instead of directly ascending, is drawn downwards and carried off by a horizontal trunk beneath the ceiling of the room below. This miracle business does away, it is true, with the objection on the score of obstruction to supervision, but it lands us in increased expense and produces an arrangement which does not always work efficiently when the fire is first lighted. In these days, when every penny has to be saved, the extra brickwork involved by the construction of fireplaces, breasts, and flues is in itself a very strong condemnation of the whole arrangement, wherever placed, and if as a consequence of its introduction we also lose a window or a bed-space, that condemnation is very seriously enhanced.

It is of course a fact that these items of initial expense are by no means the whole argument against the ward fireplace. The coal question weighs heavily with all who have to consider the burden of running expenses. Apart from this, there comes in also the problem of labour. Every grate has to be cleaned, every fire has to be laid and lit, every scuttle has to be filled with coal, and every hundredweight of coal has to be carried from cellar to ward. The labour trouble will not get easier in the near future. If it becomes simplified, which is not likely, by a re-awakened appreciation of the joys of domestic service, it will still not become cheaper.

And so, not without tears, we conclude that the coal-fire of the wards is doomed.

Need we grieve unduly? It is often urged that the heat supplied by radiators is not so wholesome, that it is smelly and oppressive, and that it does not conduce to such efficient ventilation. The answer to these objections is undoubtedly that central heating, like any other form of heating, needs attention and regulation. An overheated building will be oppressive whether it is heated by steam, hot water, or blazing coal. If radiators are dirty and dusty, they will smell of roasted dust and frizzling dirt. Obviously they must be easily cleanable and constantly cleaned. As to oppressiveness, no room need feel oppressive heat if fresh air is admitted, and in England, at least, we are agreed that the admission of fresh air is essential to health. This article was not intended to deal with the wider subject of central heating in general, but it may be as well to mention in this connection that all systems of heating by heated air, rather than by heated water, are open to the grave objection that they depend upon the use of air-flues, used either for inlet or outlet, or both. Flues of this description have been largely employed both in plenum systems and exhaust systems. But they are invariably bad in principle, because these flues cannot possibly be properly cleaned. They are vehicles for the transmission and cultivation of germs.

To return finally to the open fire: we say good-bye to it with a pang, but it is difficult to see how any architect pledged to advise a committee on the best use of their money in days of great financial, economic, and social stress, can possibly advise the retention in new wards of a survival which costs so much in material space, labour, and hard cash.

**Previous articles appeared on Jan. 25, Feb. 8, March 1, 15, 29, April 19, May 17, June 14, July 19, and Aug. 23, p. 523.**

